# Evaluation of the relationship between endolymphatic hydrops and hearing loss in Meniere’s disease based on three-dimensional real inversion recovery sequence

**DOI:** 10.1016/j.bjorl.2023.101314

**Published:** 2023-08-28

**Authors:** Yan Huang, Pengfei Zhao, Zhihao Han, Jing Xie, Yuhe Liu, Shusheng Gong, Zhenchang Wang

**Affiliations:** a Capital Medical University, Beijing Friendship Hospital, Department of Radiology, Beijing, China; b Qingdao University, Weihai Central Hospital, Department of Imaging, Weihai, China; c Capital Medical University, Beijing Friendship Hospital, Department of Otolaryngology, Head and Neck Surgery, Beijing, China

**Keywords:** Meniere's disease, Magnetic resonance imaging, Endolymphatic hydrops, Hearing loss

## Abstract

**Objectives:**

The degree of endolymphatic hydrops in Meniere's disease may be related to hearing loss. However, the results of prior studies have been inconsistent. We aimed to investigate the relationship between endolymphatic hydrops and hearing loss characteristics in Meniere’s disease.

**Methods:**

This study included 54 patients (62 ears) with Meniere’s disease. Patients underwent three-dimensional real inversion recovery sequences for magnetic resonance imaging and pure-tone audiometry. Endolymphatic hydrops were assessed according to Gurkov’s criteria (2011). Correlations between different degrees of endolymphatic hydrops and pure-tone audiometry, as well as staging, were analysed.

**Results:**

Pure tone audiometry and staging were higher for vestibular endolymphatic hydrops complicated by cochlear ones than isolated cochlear or vestibular hydrops (both *p* < 0.05). There was no significant correlation between vestibular endolymphatic hydrops and pure-tonal audiometry or staging (all *p* > 0.05). The degree of hydrops in the middle turn was correlated with the mid-frequency hearing threshold (*p* < 0.05). The degree of cochlear hydrops was correlated with the audiometry, low-frequency hearing thresholds, mid-frequency hearing thresholds and staging (*p* < 0.01).

**Conclusion:**

The types and sites of endolymphatic hydrops based on a 3D-real inversion recovery sequence can be used to indicate the degree of hearing loss in patients with Meniere’s disease.

**Level of evidence:**

Level III.

## Highlights

• Endolymphatic Hydrops (EH) in Meniere's Disease (MD) may be related to hearing loss.
• We investigated the relationship between EH and hearing loss characteristics in MD.
• Type and site of EH based real IR sequence can indicate the degree of hearing loss in MD.

## Introduction

Meniere's Disease (MD) is an inner ear disorder of unknown origin, with Endolymphatic Hydrops (EH) as the main pathological feature.^1^ It is typically characterized by recurrent spontaneous vertigo, tinnitus, and fluctuating hearing loss. EH can occur in the cochlea and vestibule or affect both structures. Damage to the organ of Corti in the endolymph of the cochlea may lead to hearing loss. Different parts of the cochlea have different characteristic frequencies: the cochlear basal turn and the cochlear apical turn are responsible for high- and low-frequency sound-signal encoding, respectively.^2^

The diagnosis of MD has traditionally relied on medical history, specialist examinations, and audiological findings.^3^ Magnetic Resonance Imaging (MRI) examinations have been used to exclude other etiologies in patients with suspected MD. In 2007, Nakashima et al. visualized the degree of EH using MRI after the injection of Gd contrast agents via the tympanic ventricle.^3^ This procedure was subsequently deemed unique in non-invasively visualizing EH, thereby becoming an essential tool in the diagnosis of MD. In 2017, the Japanese Society for Equilibrium Research proposed a new type of “certain” MD based on “definite” MD, which is determined by the presence of EH in the inner ear on MRI after the injection of Gd contrast agents.^4^

The degree of EH as revealed by inner ear MRI after the administration of Gd correlates with the severity of hearing loss. However, the results of studies have been inconsistent due to the varied techniques of inner ear MRI and the assessment criteria used to determine the degree of EH, as well as inter-subject variation. Three-dimensional Fluid-Attenuated Inversion Recovery (3D-FLAIR) sequences are mostly used for inner ear MRI after the administration of Gd contrast agents, but the use of Three-Dimensional real Inversion Recovery (3D-real IR) sequences has gradually increased. An advantage of 3D-real IR over 3D-FLAIR is the high signal-to-noise ratio and contrast-to-noise ratio,^5^ which facilitate further differentiation of the internal and external lymph from the surrounding bone signal.^6^, ^7^ Among the various criteria that exist for grading EH, the criteria proposed by Nakashima et al.^3^ in 2009 and Gürkov et al.^8^ in 2011 are commonly used. The latter classifies imaging signs based on typical dimensions in the cross-sectional plane and has a better inter-observer agreement.^9^

This study used 3D-real IR sequences for Gd-enhanced posterior inner ear MRI, and the assessment criteria proposed by Gürkov et al.^8^ to investigate the relationship between EH and hearing loss in MD.

## Methods

### Patients

A total of 54 patients who attended a university hospital in China between April 2019 and May 2023 and were diagnosed with MD based on the 2015 guidelines and had not received previous treatment were included.^10^ Among them, 21 were men and 33 were women; the mean age was 56.76 ± 10.23 (range, 16–75) years. There were 44 cases of unilateral MD and 10 cases of bilateral MD. Five ears with poor image development were excluded; thus, a total of 62 affected ears and 41 healthy ears were included. The study protocol was approved by the appropriate ethics committee, and all patients signed the informed consent form.

### Pure-tone audiometry

All patients underwent Pure-Tone Audiometry (PTA) within 1 week of MRI. PTA was performed in a standard sound-isolation room with background noise of <30 dB(A). This test was conducted using the Otometrics audiometer (Astera), which measured the conventional frequency thresholds of 250–8000 Hz for air conduction and 500–4000 Hz for bone conduction. According to the PTA grading criteria of the 1997 World Health Group, the average thresholds of binaural air conduction at 500, 1000, 2000, and 4000 Hz were calculated as the average hearing threshold (PTA 500–4000 Hz). The average thresholds at 1000 and 2000 Hz were used as the mid-frequency hearing threshold (PTA 1000–2000 Hz) and those at 4000 and 8000 Hz were used as the high-frequency hearing threshold (PTA 4000–8000 Hz). MD staging was obtained by calculating the average hearing thresholds at 500, 1000, 2000, and 3000 Hz^11^; both ears were evaluated separately for patients with bilateral MD. The average hearing thresholds for stages 1, 2, 3, and 4 were ≤25, 26–40, 41–70, and >70 dBHL, respectively.

## MRI

The patient was placed in the prone position with the head tilted to the side, and the external auditory canal was locally disinfected. The tympanic membrane surface was subsequently anesthetized using tetracaine hydrochloride and punctured at the anterior lower quadrant; 0.5 mL of a Gd contrast agent (gadopentetate and saline mixed at a ratio of 1:7) was then injected into the tympanic chamber. This procedure was repeated on the other ear. After the injection, the patient was instructed to lie in the supine position with the affected ear facing upward for approximately 30 min, with minimal talking and swallowing. MRI was performed 24 h later. The specific parameters of the 3D-real IR sequence were as follows: echo time 187 ms, repetition time 6000 ms, Inversion Time (TI) 1730 ms, bandwidth 501 Hz/Px, echo train length 124, flip angle 120°, field-of-view 200 × 200 mm^2^, matrix 384 × 384, slice thickness 0.8 mm, PAT2, and scan time 6 min 38 s.

### Evaluation of EH

**Figure 1:**
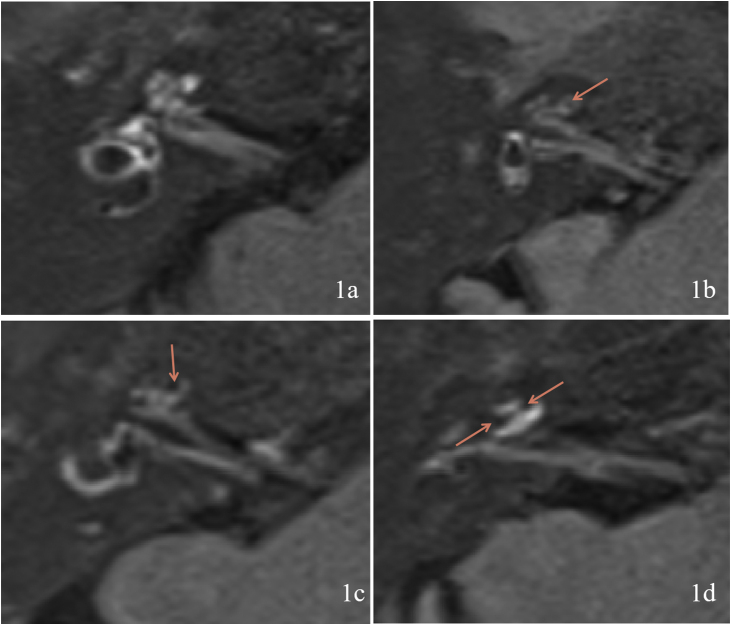
Degree of cochlear endolymphatic hydrops. (1a) Right cochlear Endolymphatic Hydrops (EH) is grade 0. (1b) Right cochlear EH is grade 1. (1c) Right cochlear EH is grade 2. (1d) Right cochlear EH is grade 3. Orange arrow shows the area of EH.

**Figure 2:**
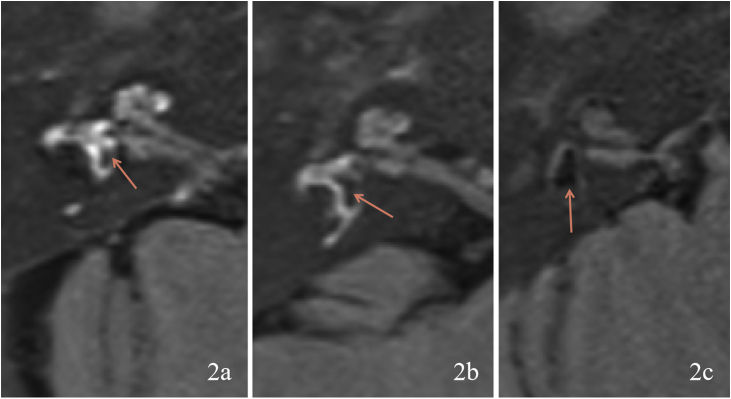
Degree of vestibular endolymphatic hydrops. (2a) Right vestibular Endolymphatic Hydrops (EH) is grade 0. (2b) Right vestibular EH is grade 1. (2c) Right vestibular EH is grade 2. Orange arrow shows the area of EH.

The MRI results were classified according to the degree of EH using the criteria described by Gürkov et al.^8^ in 2011. Four scales were used for cochlear EH: Scale 0 (none hydrops, no enlargement of the endolymphatic space is noted in the cochlea and vestibule, and the perilymphatic space is clearly visible) (Fig. 1a); Scale 1 (mild hydrops, the endolymphatic space is enlarged with hypointensity bulging into the perilymphatic space with hyperintensity) (Fig. 1b); Scale 2 (marked hydrops, the endolymphatic space is enlarged, the scala media is convex to the scala vestibuli, and the perilymphatic space is semicircular) (Fig. 1c); and Scale 3 (extreme hydrops, the endolymphatic space is severely dilated, the scala media continues to be convex to the scala vestibuli, and the perilymphatic space is compressed into a flattened line) (Fig. 1d). Vestibular EH was classified into three degrees by calculating the ratio of the area of the endolymphatic space to the total area of the endolymphatic and perilymphatic spaces (R-value), with R ≤ 1/3 for Grade 0 (no hydrops) (Fig. 2a), 1/3 < R < 1/2 for Grade 1 (mild hydrops) (Fig. 2b), and R ≥ 1/2 for Grade 2 (significant hydrops) (Fig. 2c). All imaging results were evaluated by two specialized radiologists. Inconsistent results were resolved by consulting a senior radiologist.

### Statistical analysis

Measurement data and count data are reported as the mean ± standard deviation and rate (%), respectively. Fisher's exact test was used to analyze EH in different locations within the cochlea. The *t*-test was used to compare the mean hearing thresholds at different hydrops sites. The Mann–Whitney *U* test was used to compare the clinical staging of different sites. The correlations among the degree of EH, PTA, and the clinical stage were analyzed using the Spearman rank correlation test; *p* < 0.05 was considered to indicate statistical significance. All data were analyzed using SPSS 25.0.

## Results

### PTA and staging

The PTA 500–4000, 250–500, 1000–2000, and 4000–8000 Hz values for the 62 affected ears were 58.97 ± 18.15, 59.48 ± 18.27, 57.34 ± 20.21, and 64.84 ± 18.95 dBHL, respectively. The staging results for the 62 affected ears were as follows: 5 ears in stage 1, 6 ears in stage 2, 36 ears in stage 3, and 15 ears in stage 4.

### Comparison of PTA and staging at different EH sites

There were 2 ears with no hydrops, 8 ears with isolated cochlear EH, 3 ears with isolated vestibular EH, and 49 ears with cochlear and vestibular EH. The PTA 500–4000 Hz value was 61.25 ± 17.79 dBHL for the cochlear and vestibular EH group, 48.52 ± 17.73 dBHL for the isolated cochlear or vestibular EH group, and 54.38 ± 2.65 dBHL for the no hydrops group. The PTA of the cochlear and vestibular EH group was higher than that of the isolated cochlear or vestibular EH group (*t* = 2.150, *p* < 0.05) but did not significantly differ from that of the no hydrops group (*t* = −0.450, *p* > 0.05). Staging in the isolated cochlear or vestibular EH group was longer than that in the cochlear and vestibular EH group (*Z* = −2.239, *p* < 0.05) but did not significantly differ from that in the no hydrops group (*Z* = −0.972, *p* > 0.05).

### Correlation of the degree of vestibular EH with PTA and staging

In terms of the degree of vestibular EH in the 62 affected ears, 10 ears were grade 0, 29 ears were grade 1, and 23 ears were grade 2 (Table 1). There was no significant correlation between the grading of vestibular EH in the affected ear and both PTA 500–4000 Hz and clinical stage (all *p* > 0.05). There was also no significant correlation between the vestibular EH of the affected ear and the PTA 250–500, 1000–2000, and PTA 4000–8000 Hz values (all *p* > 0.05).

**Table 1:** Degree of endolymphatic hydrops in different parts of the inner ear.

Distinct regions	Grade 0 (n)	Grade 1 (n)	Grade 2 (n)	Grade 3 (n)
Vestibule	10	29	23	
Cochlea	5	14	36	7
Cochlear middle turn	5	17	37	3
Cochlear basal turn	6	24	27	5

### Correlation of the degree of cochlear EH with PTA and staging

**Figure 3:**
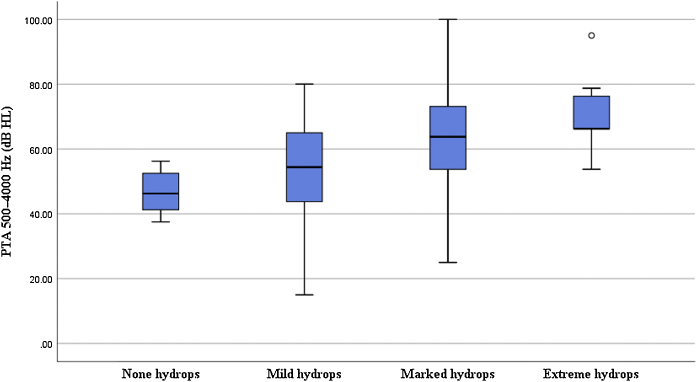
Correlation between the degree of cochlear endolymphatic hydrops and pure-tone audiometry.

**Figure 4:**
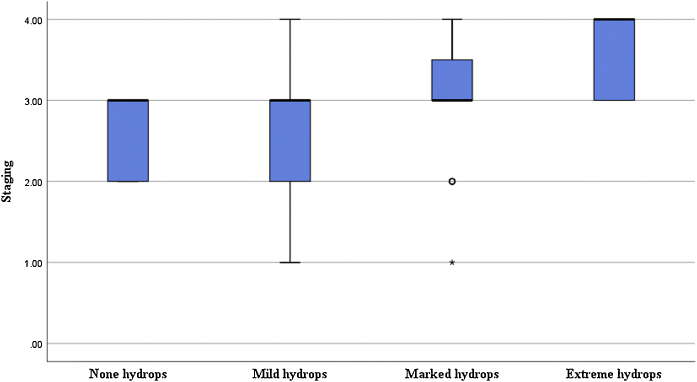
Correlation between the degree of cochlear endolymphatic hydrops and disease staging.

In terms of the degree of cochlear EH in the 62 affected ears, 5, 14, 36, and 7 ears were classified as grades 0, 1, 2, and 3, respectively (Table 1). The degree of cochlear EH in the affected ear was significantly correlated with the PTA 500–4000 Hz value (*r* = 0.417, *p* = 0.001; Fig. 3). The degree of cochlear EH in the affected ear was positively correlated with the PTA 250–500 and 1000–2000 Hz values (*r*-values of 0.479 and 0.426, respectively; both *p* < 0.01). There was no significant correlation between the degree of cochlear EH in the affected ear and the PTA 4000–8000 Hz value (*p* > 0.05). However, the degree of cochlear EH in the affected ear was significantly correlated with staging (*r* = 0.325, *p* = 0.010; Fig. 4).

### Correlation between the degree of EH and PTA at different cochlear sites

The cochlear apical turn EH rate was 93.55% (58/62), the middle turn EH rate was 91.94% (57/62), and the basal turn EH rate was 90.32% (56/62); the differences among these three rates were not significant (*p* > 0.05, Table 2). The EH grades for the cochlear basal turn were as follows: no hydrops (6 ears), grade 1 (24 ears), grade 2 (27 ears), and grade 3 (5 ears). For the cochlear middle turn, 5 ears had no hydrops, 17 ears were grade 1, 37 ears were grade 2, and 3 ears were grade 3. The PTA 1000–2000 Hz value in the affected ear was significantly correlated with the degree of middle turn EH (*r* = 0.324, *p* = 0.01), while the PTA 4000–8000 Hz value in the affected ear was not significantly correlated with the degree of basal turn EH (*p* > 0.05) (Table 3).

**Table 2:** Endolymphatic hydrops in different parts of the cochlea.

Cochlear EH sites	Hydrops (n)	No hydrops (n)	Total (n)	Hydrops rate (%)
Apical turn	58	4	62	93.55
Middle turn	57	5	62	91.94
Basal turn	56	6	62	90.32

EH, endolymphatic hydrops.

**Table 3:** Correlation between the degree of endolymphatic hydrops in different parts of the cochlea and the characteristic frequency hearing threshold.

Characteristic frequency	Cochlear EH sites	*r*	p-Value
PTA_1000–2000 Hz_	Middle turn	0.324	0.001
PTA_4000–8000 Hz_	Basal turn	0.013	0.919

EH, endolymphatic hydrops.

## Discussion

MRI after administration of Gd contrast agents is a commonly employed clinical tool to assess inner ear EH. While Gd contrast agents penetrate the perilymphatic space of the inner ear, they cannot penetrate the endolymphatic space due to the blood-labyrinth barrier. Perilymphatic spaces with injected Gd contrast agents appear hyperintense, while hypointensity is observed in endolymphatic spaces without Gd contrast agents; this allows for differentiation of the endolymphatic and perilymphatic spaces, and thus the objective assessment of EH. The administration of Gd contrast agents to the inner ear involves delivery via the middle ear using intravenous injection. After Gd contrast agents are injected via a tympanic membrane puncture, they pass through the round window membrane into the perilymphatic space. Tympanic membrane puncture contrast is superior to intravenous administration.^12^ In this study, Gd contrast agents were administered via intravenous injection through a bilateral tympanic puncture. Hyperintensity was clearly visible in the perilymphatic space, with good Gd penetration.

The TI of 3D-FLAIR is set to the endolymph over the zero point, at which the endolymph and cerebrospinal fluid are suppressed and appear as a hypointense area. In contrast, the perilymph is observed as a hyperintense region on the image. Since the surrounding bone is also hypointense, it is difficult to completely distinguish it from the boundary of the endolymph. This leads to some difficulties in the determination of EH. In this study, a 3D-real IR sequence was used for real-part reconstruction, and the TI of the inversion recovery sequence was at the middle of the point at which the endolymph and perilymph crossed zero. The perilymph, surrounding bone tissue, and endolymph appeared hyperintense, isointense, and hypointense, respectively, on the image. This greatly facilitated a more accurate distinction and identification of the site and degree of EH.^6^, ^7^

The various criteria used for the identification of EH differ slightly among researchers, and an international standard is lacking. In 2009, Nakashima et al.^3^ first graded cochlear and vestibular EH by observing the position of Reissner's membrane and calculating the R-value; both cochlear and vestibular EH were classified into three grades. The 2011 criteria are based on the 2009 criteria, which classify the cochlear EH into four grades based on the upper and lower planes of the modiolus. In contrast, the criteria by Nakashima et al. are extended for vestibular EH.^3^ Bernaerts et al.^13^ classified cochlear EH into three grades and vestibular EH into four grades by visual inspection. Fang et al.^14^ added the determination of semicircular canal EH, which was evaluated more extensively. The criteria put forth by Gürkov et al.^8^ are more detailed for cochlear EH grading and show greater consistency across observers.

MD is one of the most common causes of peripheral vertigo and is often associated with recurrent fluctuating hearing loss. Hearing loss often differs in the distinct regions of the inner ear in which EH occurs. The PTA of patients with vestibular and cochlear EH was previously found to be higher than that of patients with isolated cochlear or vestibular EH^15^; this finding is supported by the results of our present study.

The association between the degree of EH and the hearing threshold has also been reported previously. Yang et al.^16^ demonstrated that the degree of both vestibular and cochlear EH in patients with MD was significantly correlated with the hearing threshold. The results of the study indicated that the degree of cochlear EH was significantly correlated with both the mean hearing threshold and disease stage, but no direct associations were noted between vestibular EH and the mean hearing threshold or disease stage. This may be because the vestibule is mainly a human balance receptor, while the cochlea is primarily responsible for sound encoding; thus, cochlear EH may have greater effects on the function of auditory hair cells.

Some studies have focused on the association between hearing thresholds in different frequency bands and the degree of effusion. For example, Zhang et al.^17^ found that low-, mid-, and high-frequency hearing thresholds correlated well with the degree of cochlear and vestibular EH. Sun et al.^18^ found that the degree of EH was significantly correlated with the low-frequency hearing threshold and disease stage. Shi et al.^19^ also observed that the degree of cochlear and vestibular EH was significantly correlated with low-frequency hearing thresholds. Likewise, our results showed a good correlation between cochlear EH and low- and mid-frequency hearing thresholds. These findings indicate a correlation between the degree of MD-associated EH and the hearing threshold; the more severe the degree of EH, the greater the hearing loss, with the low-frequency hearing threshold being more closely related to the degree of cochlear EH.

The cochlear structure produces a specific frequency-location map due to its frequency specificity for sound encoding. Therefore, different locations of cochlear EH may cause hearing loss with different frequency characteristics. Our evaluation of hearing thresholds for different EH sites in the cochlea revealed that cochlear middle turn EH was significantly correlated with the mid-frequency hearing threshold, while no such association was found between basal turn EH and the high-frequency threshold. This may be attributed to the possibility that MD causes some alteration of the original cochlear frequency-position map after EH.^20^ In addition, the frequency range of the cochlea is wider for encoded sounds than for PTA, and the mid- and high frequencies of PTA were not exactly equivalent to the characteristic frequencies of the middle and basal turns of the cochlea. This may account for the lack of correlation between the high-frequency hearing threshold and apical turn EH. In the study, there was no significant difference in the probability of EH developing in the apical, middle, and basal turns of the cochlea, which may be due to the limited number of study participants. In contrast, some studies have reported that MD-associated EH develops in the top turn and gradually spreads to the basal turn.^21^ Overall, our results indicate that EH in different parts of the cochlea may affect hearing thresholds at characteristic frequencies, and this finding is best reflected by the relationship between the mid-frequency hearing threshold and the degree of cochlear middle turn EH.

## Limitations

This study has a few limitations. Our results showed that PTA in patients with both vestibular and cochlear EH was higher than that of patients with isolated cochlear or vestibular EH. However, as there were too few ears with isolated vestibular EH, we combined isolated vestibular EH and isolated cochlear EH into one group for analysis. There is a need for further studies with larger sample sizes, particularly for cases with no hydrops. Furthermore, due to the difficulty and subjectivity in determining the degree of apical turn EH, this study only evaluated the presence or absence of hydrops and did not grade cochlear parietal rotation EH.

## Conclusion

In summary, the greater the degree of EH in patients with MD, the greater the hearing loss. This association is particularly evident in cases of cochlear EH. A significant correlation is reported between EH in different cochlear sites and hearing loss in different frequency bands, mainly between cochlear middle turn EH and the mid-frequency PTA hearing threshold.

## Conflicts of interest

The authors declare no conflicts of interest.
